# Supplementary material for: Simultaneous Maturation of Single Chain Antibody Stability and Affinity by CHO Cell Display
Source: Bioengineering (Basel). 2022 Aug 2;9(8):360. doi: 10.3390/bioengineering9080360 (PMC9404881; doi:10.3390/bioengineering9080360)
Supplement: Supplementary file 1 [file bioengineering-09-00360-s001.zip › bioengineering-1769815-supplementary.pdf]

Fig. S1

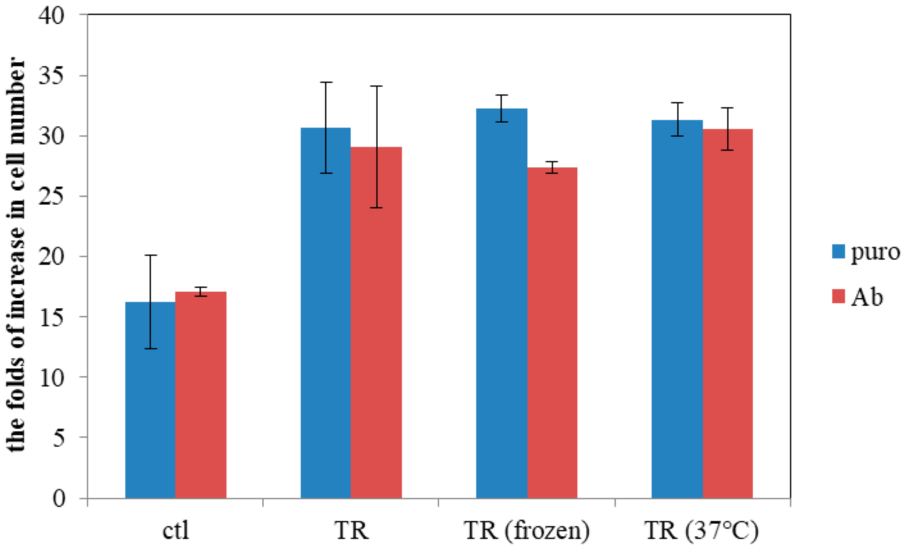

Fig. S1 The stability of thermal resistance of TR cells. The same number (3×10^5) of the four types of cells with or without displaying the antibody were cultured at 41°C for 5 days, and the total cell numbers were counted. The folds of increase in cell number were culculated. All the TR cells, etiher continuosly cultured at 41°C, after cultured at 37°C or after frozen and thawed, had obviously greater folds of cell number increase during five day culture at 41°C than the cells continuosly cultured at 37°C (Figure S1). There were much smaller differences in the cell number increase among the three types of TR cells, thus a short term culture at 37°C or a storage in liquid nitrogen is unlikely to change the thermal resistance feature of TR cells and to have negative impact on the antibody maturation." ctl:normal cells; TR: thermal resistant cells continuosly cultured at 41°C; TR (fronzen): frozen and thawed TR cells before exposure to 41°C; TR (37°C): TR cells cultured at 37°C for 4 weeks before exposure to 41°C.

Fig. S2

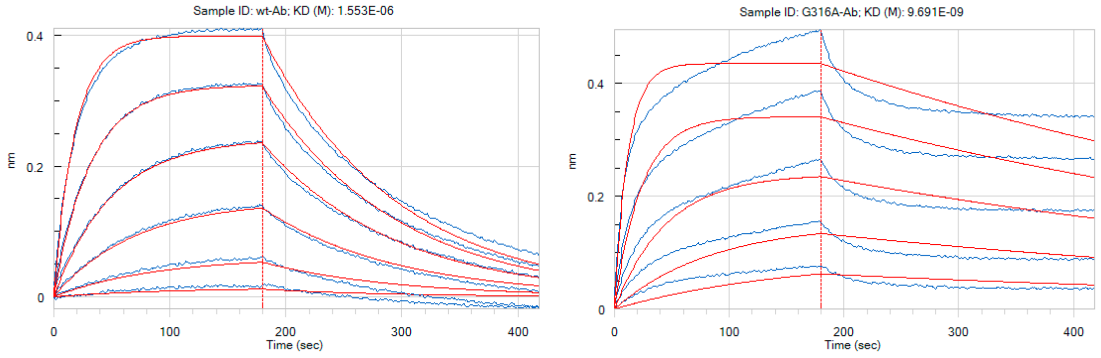

WT K_D≈1.55uM G316A K_D≈9.69nM

Fig. S2 The results of antibody affinity. The basic procedure was to immobilize the biotinylated antigen on a chip, and the antibody acted as a mobile phase to detect the affinity of the antibody. K_{on}

and K_{off} were obtained by the system software based on the combination and dissociation curves, while the KD values were calculated by K_{off} / K_{on}.

Fig. S3

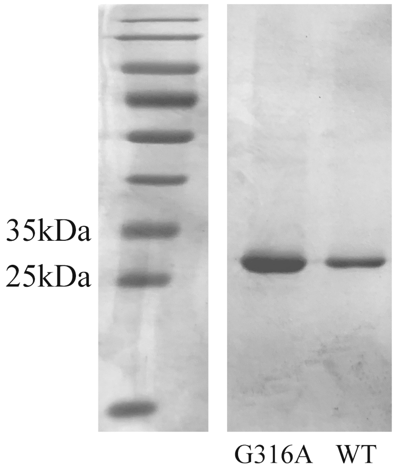

Fig. S3 The results of SDS-PAGE images. The purified proteins were analyzed by sodium dodecylsulfate, polyacrlyamide gel electrophoresis (SDS-PAGE).

Table S1 Mutations observed during the affinity maturation procedure

mutation	Cultured at 37°C<br>Incubated at 4°C		Cultured at 41°C<br>Incubated at 42°C	
	S1[a]	S2	S1	S2
G316A (HC)				20/39
C344T (HC)	6/47[b]	14/41		
C467T (LC)			2/71	
C543T (LC)	4/47	1/41		
C741T (LC)	1/47			
C344T (HC)<br>C654T (LC)		2/41		
G254C (HC)<br>C344T (HC)<br>C543T (LC)		1/41		
C268G (HC)<br>C344T (HC)<br>C543T (LC)		1/41		

CDRs are highlighted in colors (CDR1, CDR2 and CDR3).

a The first round of the single chain antibody affinity maturation after which the mutations on the antibody gene were revealed by sequencing

b The number of mutant clones / The numbers of sequenced clones

Table S2 Amino acid sequence of anti-TNFα antibody variable region

Heavy chain (HC)	QLVQSGPELKKPGETVKISCKASGYTFTNYGMNWVKQAPGKGLKWMGWINTYTGEPTYADDFKGRFAFSLETSASTAYLQINNLKNEDSATYFCAGRRSYDYDVAMDYWGQGTSVTIS
Light chain (LC)	DIVLTQSPASLAVSLGQRATISCRASESVDSYGNYFMHWYQQKPGQPPKLLIYRASNLESGIPARFSGSGSGTDFTLTINPVEADDVATYYCQQSNEEPLTFGSGTKLEIK

CDRs are highlighted in colors (CDR1, CDR2 and CDR3).

Table S3 The molecular mass of different mutations

Clone	Molecular mass (kDa)	Partial specific volume
WT	12.1	8.22%
	23.9	77.58%
	47.6	10.97%
G316A	11.8	6.19%
	24.5	80.96%
	52.5	10.43%
